# Assessing quality of life in pediatric fibrous dysplasia and McCune Albright syndrome: PEDS-QL and HADS data from the Fibrous Dysplasia Foundation Patient Registry

**DOI:** 10.1186/s41687-021-00304-2

**Published:** 2021-04-12

**Authors:** Amanda Konradi

**Affiliations:** Department of Sociology, Loyola University Maryland, 4501 Charles St., Baltimore, MD 21210 USA

**Keywords:** Fibrous dysplasia, Quality of life, Depression, Anxiety, Children, McCune Albright syndrome, PEDS-QL, HADS

## Abstract

**Purpose:**

The International FD/MAS Consortium recently encouraged using the Pediatric Quality of Life Inventory (PEDS-QL) and the Hospital Anxiety and Depression scales (HADS) in clinical care. This study examines scores on these measures among pediatric fibrous dysplasia and McCune Albright (FD/MAS) patients to initiate consideration of their use in clinical treatment.

**Methods:**

This is a retrospective analysis of pediatric data from 39 minors, ages 2–17, entered in the Fibrous Dysplasia Foundation Patient Registry from July 2016 to December 2018. Sample means and score distributions are compared to general population and chronic disease benchmarks. Associations with medical and demographic variables are also explored.

**Results:**

Mean PEDS-QL scores for children 2–7 were inconclusive in determining at risk status for impaired quality of life (QOL). Individual score distributions suggested up to half experienced extensive physical or social impairment. Means and individual score distributions for the physical and psychosocial components of the PEDS-QL for children 8–17 suggested many were at risk of impaired QOL. Over half of 13–17 year-olds met the clinical benchmark for anxiety. Older males scored better than females on the PEDS-QL and HADS. Pain frequency was associated with physical function for older children.

**Conclusions:**

Older children with FD/MAS may be more compromised in terms of psychosocial QOL than previously reported. Clinicians should be attentive to the influence of gender on QOL in older children. Online patient registries associated with rare diseases have the potential to serve as efficient and cost-effective mechanisms to jumpstart examination of new measures in consideration for clinical use.

## Plain English summary

An international group of thought leaders concerned with the care of patients with Fibrous Dysplasia/McCune Albright Syndrome (FD/MAS) recently encouraged treating physicians to use two measures to assess children’s health related quality of life (QOL), the Pediatric Quality of Life Inventory (PEDS-QL) and the Hospital Anxiety and Depression scales (HADS). To date, no one has applied these measures to the FD/MAS population. This study was conducted to initiate consideration of their use in clinical management. The study is a retrospective analysis of data from 39 children, ages 2 to 17, who are participating in the Fibrous Dysplasia Foundation Patient Registry (FDFPR). This is an online resource that enables patients to report on demographics, symptoms, surgical and medical treatments received; to complete validated psychological and social measures (including the PEDS-QL and HADS); and to answer questions about access to and satisfaction with treatment.

The scores of children with FD/MAS were compared to published scores from the general population and from children with chronic diseases. Results showed that on average, young children, 2–7, had significantly worse physical health than the general population, but similar psychological and social health. However, the PEDS-QL scores of 30–50% of these young children suggested they were at risk in some aspect of QOL. PEDS-QL score averages for older children, 8–17, and the distribution of individual scores in relation to published benchmarks, suggested half were at risk of impaired QOL. More than half the 13–17 year-olds also scored above 7 on the HADS, the published value for clinical levels of anxiety. Older males scored better than females on the PEDS-QL and the HADS. Pain frequency was associated with physical function for older children.

In sum, older children with FD/MAS may be more compromised in terms of psychosocial QOL than previously reported. Clinicians should be attentive to the potential influence of gender on QOL in older children. Online patient registries associated with rare diseases, such as the FDFPR, have the potential to serve as efficient and cost-effective mechanisms to jumpstart examination of new measures in consideration for clinical use.

## Introduction

Fibrous dysplasia (FD) is a rare congenital and genetically heterogeneous noninheritable skeletal disease in which normal bone and marrow are replaced with discrete lesions of fibrous-osseous tissue. Radiographically, the lesions resemble ground glass. FD may affect a single bone (monostotic) or multiple bones (polyostotic) and may appear in the skull, trunk and extremities. The disease is usually diagnosed in childhood. Although not routine clinical practice, a 99Tc MDP bone scan can usually identify affected skeletal regions by 5 years of age; a head CT is used to fully assess cranial involvement [1]. Molecular genetic testing can establish the presence of the mutation in lesion tissue. Monostotic FD is most common in the rib, skull and femur; polyostotic is most common in the skull, mandible, pelvic bones and femur [2]. McCune-Albright Syndrome (MAS) “is defined as the combination of FD and one or more extra skeletal feature, OR the presence of two or more extra skeletal features,” including café-au-lait skin pigmentation and endocrinopathies such as, growth hormone excess, hyperthyroidism, hypercortisolism, and renal phosphate wasting [3]. MAS is estimated to occur in 100,000 to 1,000,000 of the population and to comprise 5% of FD patients [4]. Bone lesions can cause functional problems, that vary depending on the bones affected, and pain [1]. FD may also result in problems of social interaction: too much attention, the wrong kinds of attention, or social exclusion [5].

The health related quality of life (QOL) of FD/MAS patients is a growing concern of medical research [5–11]. Investigators at the US National Institutes of Health (NIH), led by Dr. Michael T. Collins, explored QOL in Fibrous Dysplasia / McCune Albright Syndrome (FD/MAS) patients 14 and older with the SF-36, a self-report measure, which has 8 domains: physical functioning, role limitations due to physical problems, bodily pain, general health, energy/fatigue (also called vitality), social functioning, limitations on role performance due to emotional problems, and mental health. They measured the QOL of children diagnosed with FD with a parent proxy, the Child Health Questionnaire for children ages 7 to 13.

Collins and colleagues found that FD/MAS patients 14 and older exhibited physical QOL outcomes that were significantly lower (worse) than the general population but social-psychological measures that were similar [6]. Parent proxies for children 7 to 13 yielded scores for physical function, general health and bodily pain that were significantly lower than the general population, but social-psychological scores that were similar. In terms of physical function, children with FD were like children with asthma and rheumatoid arthritis. Children with FD had similar social-psychological scores to children with asthma, but significantly lower scores than those with rheumatoid arthritis [6]. Collins and colleagues found that children and adults with greater bone disease had poorer physical function [6, 12]. Using the Brief Pain Inventory (BPI), a self-report measure, they found that adults and children had greater pain than the general population, that children with FD reported less pain than adults, and that reported pain did not correlate with skeletal disease burden [7].

Recently published best practice guidelines for FD care from the International FD/MAS Consortium endorsed the evaluation of QOL in FD/MAS, encouraging the use of specific measures, among them the Pediatric Quality of Life Inventory (PEDS-QL) for children and the Hospital Anxiety and Depression Scale (HADS) [3].

The purpose of this descriptive study is to analyze available pediatric data from the Fibrous Dysplasia Foundation Patient Registry (FDFPR), an online self-report portal that incorporates these two measures, to initiate consideration of their use in clinical treatment of FD/MAS.

## Methods

### Study population - the fibrous Dysplasia Foundation patient registry

In June 2016, the Fibrous Dysplasia Foundation (FDF, now FD/MAS Alliance, https:fdmasalliance.org), a nonprofit patient advocacy organization, opened a patient registry (FDFPR, www.fdmasregistry.org/) for individuals with fibrous dysplasia and McCune Albright Syndrome, to facilitate research into this rare disease. The FDFPR consists of a battery of online surveys about the extent of a respondent’s lesions and, if relevant, endocrine involvement, symptoms, surgical and medical treatments received, psychological and social measures, demographics, and questions about access to and satisfaction with treatment. The patient population consists of individuals recruited through direct email, clinicians, the Foundation website and newsletters, patient and family conferences, two FD patient-administered Facebook groups, and Twitter.

The study is a secondary analysis of deidentified FDFPR data. The sample consists of 39 minor patients ages 2–17, about whom demographic, bone involvement and diagnosis information was known, and relevant QOL measures were completed. FDFPR participants who did not attempt to complete the PEDS-QL or HADS, if age appropriate, were excluded. In this text, “participant” refers to the individual with FD/MAS about whom data was collected, although parents/guardians completed some of the data entry. “Self-report” refers to information specifically entered by the minor. “Parent proxy” refers to data entered by the parent/guardian about the participant.

### Measures

The PEDS-QL is a generic health related QOL measure consisting of 4 core scales, physical function (8 items), emotional function (5 items), social function (5 items) and school function (5 items) that is intended for use in healthy and patient populations. Respondents are asked to recall the last month and indicate how frequently - from never to almost always - they have experienced specific phenomena. Item responses (0–100) are averaged to form total and core scores; higher scores indicate higher functioning [13, 14]. If more than 50% of a scale has been answered, missing scores may be derived from the average of existing scores.

Self-report versions of the PEDS-QL are available for ages 5–7, 8–12, 13–17 and comparable parent proxy versions are available from ages 2–17. Question wording is sustained between the versions so that responses may be compared across ages. This is desirable from a clinical perspective. Research shows alignment between self-reports and proxy reports is moderate to good, allowing for either form to be used in clinical assessment [15–17]. The school function scale should only be used for descriptive or exploratory analysis with children 2–7 [13]. The PEDS-QL is responsive to clinical improvement across the physical and psychosocial scores as well as the total score [18]. Furlong and colleagues recommended the PEDS-QL for use as the generic health profile for pediatric orthopedic research [19].

There is not yet a consensus on the interpretation of PEDS scores. Initially, Varni and colleagues suggested that scores one standard deviation below healthy child means present “at risk status for Impaired HRQOL” (p332) [13]. Later, they presented findings of significant mean differences in the total scores of healthy children and those with chronic illnesses (for self-reports and parent proxies) [20]. Huang and colleagues found significant mean differences among parent proxy scores for children ages 2 to 7 and 8 to 18 and recommended use of separate age related clinical benchmarks for clinical follow up [21].

In the FDFPR, children 8 or older may complete the age-appropriate self-report version of the PEDS-QL (8–12, 13–18). Parents/guardians complete the proxy version for children ages 2–7 and for older children who do not complete the self-report. While either parent proxies or self-reports may be adequate for clinical assessment, they may not be aggregated for research purposes. As discussed in the analytic strategy below, self-report and parent proxy data were handled separately in this study. Score averaging was used to produce answers for three skipped questions in the data set.

The Hospital Anxiety and Depression (HADS) scale is a set of fourteen items that concern how often patients have experienced specific feelings in the past week that relate to the loss of pleasure. Answer values range from 0 to 3 and are added to produce separate anxiety and depression scores, from 0 to 21 [22, 23].

The HADS is validated on general and specific disease populations; it is used extensively in research, especially in Europe [24]. The developers have designated scores from 0 to 7 as non-clinical (normal), from 8 to 10 as mild, from 11 to 15 as moderate, and 16 or more as severe [25]. Many validation studies have affirmed the clinical cutoff of 8, but some research on specific diseases, including cancer and chronic obstructive pulmonary disease (COPD), has suggested a need for a lower benchmark [26–28].

Children aged 13 to 17 in this study completed the HADS. No data was missing.

One item from the Brief Pain Inventory concerning how often the participant experienced pain was included for all children.

Demographics included in this study were the participant’s age, gender, race, and both parent’s educational attainment. Parents/guardians were directed to reference lists of bones to indicate which were affected by FD, and asked to report the patient’s medical diagnosis, as monostotic FD, polyostotic FD or FD with McCune Albright Syndrome. Reports of monostotic and polyostotic disease were cross referenced with the bones listed and found to be consistent; reports of endocrinopathies were not cross-checked. Craniofacial disease burden was calculated from the number of zones in which FD lesions were identified in the skull, following Chen and colleagues [29].

### Analytic strategy

Univariate statistics were generated to describe the sample and combined scores on the QOL measures. Bivariate analysis was conducted to establish whether demographics were significantly associated with type of diagnosis, craniofacial involvement, and type of report (self or proxy). The sample was divided by age group, 2 to 7 and 8 to 17, and by report type to generate means for the total and core scores of the PEDS-QL. T-tests were then used to compare sample means to Varni and colleagues’ normative data for self-reports and parent proxies for the general population [13], specific chronic diseases [20], and to the age specific bench marks for parent proxy scores provided by Huang and colleagues [21]. The distribution of individual scores across benchmarks was also assessed. Mean HADS results were computed and individual scores were distributed into Snaith’s nonclinical, mild, moderate and serious categories [25]. ANOVAS were used to determine whether QOL scores were significantly associated with participant’s diagnosis type, craniofacial involvement, frequency of pain, and gender.

## Results

### Demographic and medical features of the sample (see Table 1)

The 39 participants were slightly more than half male (51.3%), overwhelmingly white (87.2%), non-Hispanic (88.2%), and living with parents who had the benefit of a college education (fathers 61.1% and mothers 65.8%). Their mean age was 9.7 years and ranged from 2 to 17. The majority had polyostotic disease (48.7%), followed by FD/MAS (30.8%), and monostotic disease (20.5%). Forty six percent of the sample reported craniofacial involvement. Thirty-eight percent of them reported craniofacial involvement in one zone, 16.7% in two zones, 16.7% in three zones and 27.8% in all five zones. Chi Square tests did not reveal significant relationships between patient demographics, and diagnosis, craniofacial-involvement, or report type.

**Table 1:** Characteristics of the Study Population

Characteristics and N	Values	Frequency	Percentage
Gender (*N* = 39)	male	20	51
	female	19	49
Ethnicity (*N* = 34)	Hispanic/Latino	2	6
	Non-Hispanic	30	88
	Ashkenazi Jewish	2	6
Race (*N* = 39)	White	34	87
	Black and other	5	13
Mother’s education (*N* = 38)	High school/GED or less	7	18
	Some college or trade school	6	16
	College of more	25	66
Father’s education (*N* = 36)	High school/GED or less	5	14
	Some college or trade school	9	25
	College of more	22	61
Diagnosis (*N* = 39)	monostotic FD	8	21
	polyostotic FD	19	49
	FD and McCune Albright	12	31
Craniofacial FD (*N* = 38)	no	21	54
	yes	18	46
CF Zone 1 (*N* = 18)	no	6	33
	yes	12	67
CF Zone 2 (*N* = 18)	no	7	39
	yes	11	61
CF Zone 3 (*N* = 18)	no	7	39
	yes	11	61
CF Zone 4 (*N* = 18)	no	10	56
	yes	8	44
CF Zone 5 (*N* = 18)	no	13	72
	yes	5	28
CF Disease Burden (*N* = 18)	1	7	39
	2	3	17
	3	3	17
	4	0	0
	5	5	28

### PEDS-QL

**Fig. 1:**
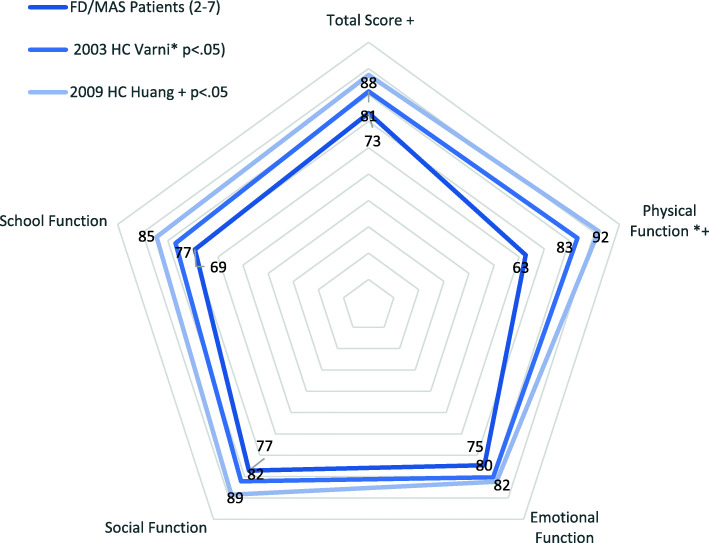
PEDS-QL parent proxy ages 2–7 compared to Healthy Child norms from Varni (2003) and Huang (2009). The pentagram vertices indicate sample total and core means in relation to the two benchmarks. Significant differences (<.05) are noted

Means were calculated for the full set of parent proxies, which included some older children, and separately for children ages 2–7. A t-test of means revealed no significant differences for any of the core scores, so the remainder of the parent proxy analysis was conducted with 14 children ages 2–7, who could be compared to the age graded cutoffs calculated by Huang and colleagues [21] (See Fig. 1). All mean core scores for participants ages 2–7 were below (worse than) the 2003 Health Child (203HC) norms [13], but only physical function was significantly so (2.653, *p* = .02; 2007, − 2.81, *p* = .015). Mean total scores for participants ages 2- to 7 were less than one standard deviation below Varni and colleagues’ 2003HC norm (not meeting their criteria for “risk” of impaired health related QOL) [13]. Participant’s mean total scores and physical function scores were significantly lower than Huang and colleagues’ mean scores for Healthy Children ages 2–7 [21].

Close to half of the 2–7 year-olds had total PEDS-QL scores and physical function scores more than one standard deviation below Varni and colleagues 2003HC means (46% and 50%, respectively), while almost a third (31%) scored more than a standard deviation below healthy norms for the psychosocial domains [13] (See Table 2). More than half of the children also scored below benchmark values designated as “serious” by Huang and colleagues for the physical, emotional and social domains [21].

**Table 2:** Distribution of Total and Core Scores on the PEDS-QL in Relation to Published Benchmarks

**PEDS-QL parent proxy scores for participants aged 2–7 compared to Varni (2003) Healthy Child mean and one standard deviation (at risk) benchmark (*N*** **= 13)**					
		% Scoring above 2003 HC Mean	% Scoring less than 1 standard deviation below HC Mean	% Scoring more than 1 standard deviation below HC Mean	
Total Score	> 81.34, 81.34, 68.18	38	15	46	
Physical Function	> 83.26, 83.26, 69.38	29	21	50	
Emotional Function	> 80.28, 80.28, 61.64	36	43	21	
Social Function	> 82.15, 82.15, 64.74	43	29	29	
School Function	> 76.91, 76.19, 59.98	46	23	31	
**PEDS-QL parent proxy scores for participants aged 2–7 compared to Huang (2009) benchmarks (*N*** **= 13)**					
	Cutoff Scores	% Scoring Above Mild benchmark	% Scoring Below Mild benchmark	% Scoring Below Moderate benchmark	% Scoring Below Serious benchmark
Total Score	> 83, 83, 79, 77	38	0	15	46
Physical Function	> 91, 91, 88, 84	14	14	0	72
Emotional Function	> 75, 75, 75, 75	43	0	0	57
Social Function	> 85, 85, 80, 80	43	0	0	57
School Function	> 75, 75, 75, 67	0	46	8	46
**PEDS-QL self-report scores for participants aged 8–17 compared to Varni (2003) Healthy Child mean and one standard deviation (at risk) benchmark (*N*** **= 18)**					
		% Scoring above 2003 HC Mean	% Scoring less than 1 standard deviation below HC Mean	% Scoring more than 1 standard deviation below HC Mean	
Total Score	> 82.87, 82.87, 69.71	11	28	61	
Physical Function	> 86.86, 86.86, 72.98	22	22	56	
Emotional Function	> 78.21, 78.21, 59.57	17	22	61	
Social Function	> 84.04, 84.04, 66.61	28	22	50	
School Function	> 79.92, 79.92, 62.99	22	17	61	

**Fig. 2:**
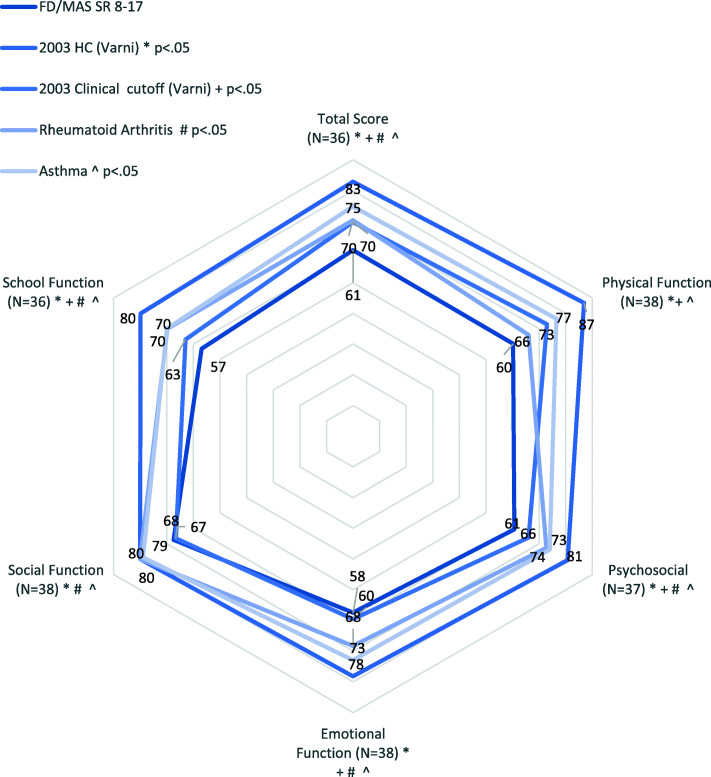
PEDS-QL Self Reports Aged 8–17 Compared to Healthy Child and Chronic Illness Norms from Varni (2003 and 2007). The pentagram vertices indicate sample total and core means in relation to the two benchmarks. Significant differences (<.05) are noted

Results of self-reports for 18 children ages 8–17 are in Fig. 2 and Table 3. All mean core scores were significantly below Varni and colleagues’ 2003HC norms for self-reports and, other than social function, also more than one standard deviation below them (meeting criteria for “risk” of impaired health related QOL) [13]. Half or more of the FD patients aged 8–17 scored more than one standard deviation below Varni and colleagues 2003HC core means, suggesting risk of impaired QOL [13].

**Table 3:** Hospital Anxiety and Depression Scale Individual Score Distribution Ages 13–17

Variable	Values	Frequency	%
Anxiety	Non-clinical (0–7)	8	42
	Mild (8–10)	8	42
	Moderate (11–14)	3	16
	Serious (15–21)	0	0
		*N* = 19	
Depression	Non-clinical (0–7)	18	95
	Mild (8–10)	1	5
	Moderate (11–14)	0	0
	Serious (15–21)	0	0
		*N* = 19	

Older children (8–17) with FD/MAS scored similarly to pediatric patients with Rheumatoid arthritis [20] on physical function, but significantly below them on all other aspects of quality of life (See Table 3). All their mean core scores were also significantly below pediatric patients with Asthma [20] (See Fig. 2).

### Hospital anxiety and depression scale

HADS data was available for nineteen children ages 13 to 17 (See Table 3). Mean scores for the anxiety and depression scales were 7.37 (SD 3.96) and 2.68 (SD 2.45), respectively, less than 8, the benchmark for clinical “caseness” and recommended follow up. Yet, among the sample, almost 58% of the participants had anxiety scores of 8 or above, meeting criteria for follow up (15.8% in the moderate category and 42.1% in the mild category). Only one adolescent (5.3%) had a depression score above 7.

### Bivariate analysis

Analysis of variance did not reveal a consistent directional association or significant relationship between PEDS-QL total or core scores and patient diagnosis (monostotic, polyostotic, or FD with MAS) for children 2–7 or 8–17. Similarly, craniofacial involvement was not significantly related to total scores for either age group. Neither diagnosis nor craniofacial involvement was significantly associated with adolescent’s (13–17) anxiety or depression scores. Power in these analyses was low.

Reported frequency of pain correlated significantly and negatively with the PEDS-QL physical function score for children 8–17 (*R* = -.536, *p* = .026). However, no significant relationships were found between pain and other PEDS-QL core scores for children 2–7 or 8–17. Pain also did not correlate significantly with the anxiety and depression scores of adolescents.

ANOVAs did not reveal significant gender differences in the PEDS-QL total score or core scores of participants aged 2–7 (See Table 4). Males 8–17 scored higher (better) than females 8–17 on the total score and all core scores, of which the difference in physical function was significant (*p* = .036,). Mean HADS scores for females aged 13–17 were higher (worse) than means scores for males for both anxiety (9.13 (SD3.36) v 6.09 (SD4.01)) and depression (4.13 (SD2.36) vs 1.62 (SD2.01). The difference for depression was significant (F = 6.140, *p* = .024).

**Table 4:** Gender x PEDS-QL and HADS scores ANOVA

PEDS-QL Elements	Patients Ages 2–7 (proxy)					
	Males	Females	F	*p* value	Obs. Power	R^2^
Total Score	73	73	.002	.969	.050	.000
Physical Function	69	56	.612	.450	.111	.053
Emotional Function	82	71	1.422	.256	.196	.106
Social Function	74	79	.147	.710	.164	.012
School Function	68	70	.013	.910	.051	.001
	Patients Ages 8–17 (self-report)					
	Males	Females	F	*p* value	Power	R^2^
Total Score	79	48	4.187	.110	.348	.511
Physical Function	70	43	5.295	.036	.576	.261
Emotional Function	62	50	1.619	.221	.223	.092
Social Function	72	62	1.698	.206	.238	.072
School Function	39	54	.130	.723	.063	.008
HADS Elements	Patients Ages 13–17					
	Males	Females	F	*p* value	Power	R^2^
Anxiety	6.09	9.13	3.023	.100	.375	.150
Depression	1.63	4.13	6.140	.024	.647	.265

## Discussion

The present study is the first to examine quality of life of children with FD /MAS using the PEDS-QL and HADS measures. Results from a natural history study conducted by Collins and colleagues at NIH, involving other measures, can fruitfully be contrasted with it. Young children (2–7) with FD/MAS in this study resembled those in the natural history study, scoring below healthy norms for physical function, but similarly to healthy children on psycho-social elements [6]. Older children in this study (8–17) differed from those in the NIH study, with mean scores indicating substantially compromised QOL relative to healthy children. Yet, like the children in the natural history study, the older pediatric FD/MAS patients in this study scored similarly to children with pediatric Rheumatoid Arthritis on physical function, but comparatively worse on psychosocial components of QOL [6]. Unlike the children studied at NIH, the scores of older FD/MAS patients indicated worse QOL than pediatric patients with Asthma [6]. Worse scores on psychosocial components of the PEDS-QL in adolescents with FD/MAS than children with other chronic diseases may reflect the difficulty of managing physical symptoms of a rare condition that sets them outside age group norms without a supportive identity community [30]. Craniofacial differences, in particular, may result in difficult interactions, negative emotions, and a need to manage a stigmatic identity [31].

Collins and colleagues developed the natural history study sample from online recruitment and individuals referred to the National Institutes of Health. Participation was by invitation and reflected investigators’ interests in MAS and endocrinopathies as well as bone lesions. This sample was comprised of all who volunteered. Thus, some differences described above and below may reflect differences in the samples.

These results suggest that anxiety at a level of clinical concern is common among adolescent patients (13–17) with FD/MAS, while depression is not. Compared to adolescents (ages 10–19) with bladder cancer, FD/MAS patients were more anxious (56% versus 27%), but had a similar rate of depression (5.9% versus 5%) [32]. Compared to a sample of adults with FD/MAS (unpublished results), these adolescents had lower mean scores for anxiety (7.37 v 8.49) and depression (2.68 v 5.66). However, the percentage of individuals in the adult and pediatric samples who scored above 7, indicating at least mild symptoms of anxiety were similar (56% v 55.6%, respectively). Far fewer adolescents than adults scored above 7 for depression (5% vs 33.6%, respectively). Considered in light of the established association between adolescent social anxiety and later depression and also greater severity of depression, a higher level of depression among adult FD/MAS patients supports further clinical assessment of adolescents with elevated anxiety scores [33]. Anxiety may not cause depression, but its presence in adolescence may indicate a greater propensity to develop the condition [34].

Collins and colleagues found that among children reported pain was not associated with psychosocial measures but was associated with physical function [7]. This study affirmed the lack of association with psychosocial measures and frequency of reported pain in all children, but found that the association of physical function and pain varied by age group. Variation in pain results may reflect use of pain frequency in contrast to pain intensity or differences in the nature of the reports used in this study, self-reports for older children and proxies for younger children. Alternatively, progression of disease may result in more frequent pain, or older children may experience different kinds of pain, including in the craniofacial region which is difficult to treat.

Research conducted in the Netherlands found a significant association between QOL measures and severity of diagnosis and craniofacial involvement in adults [11]. In contrast, this pediatric sample found neither was independently related to PEDS-QL or HADS scores. Lack of a relationship may reflect the sample size (power estimations were low) or distribution of FD/MAS types and, with respect to psychosocial scores, the distribution of craniofacial lesions in zones of the skull. It is also possible that the PEDS-QL does not capture unique features of the experience of living with FD/MAS and is inadequate to ascertain clinically important differences [35]. For example, it lacks physical function questions that address craniofacial matters and the psychosocial questions do not address stigma. Lesions that are not visible, due to their location, their inward expansion, or the extent of their development may pose less of an impediment to interaction. The age distribution in the sample may also matter to the extent that craniofacial differences among, elementary school aged children are less often negatively marked by peers than those of middle and high school aged children.

Validation studies of the PEDS-QL conducted by Varni and colleagues did not reveal gender sensitivity [13, 15]. In contrast, among healthy Norwegian adolescents, females scored lower than males on physical function, emotional function, and the total score [36]. This study also found gender differences in QOL scores among older children, with females scoring significantly below males on physical function domain of the PEDS-QL and lower on other psychosocial measures.

Some validation studies report no significant gender differences in adult HADS scores [37, 38]. Others report adult females score significantly higher on anxiety and depression than males [39, 40], males score higher than females [27], and that the HADS overestimates depression among women [41]. Two studies have reported significant gender differences in HADS scores among adolescents and young adults [42, 43], with females exhibiting higher levels of distress. This study also found adolescent females scored significantly higher than adolescent males on depression. Observed power of this analysis was comparatively high.

A gender difference in QOL among the older children may arise from their growing understanding of and adherence to gender norms. Adolescent boys may feel a need to deny physical limitations and/or the existence of negative emotions, resulting in higher scores. The loss of confidence and self-esteem that psychologists have noted adolescent girls experience in comparison to boys may contribute to their comparatively lower scores [42, 44]. It is also possible that girls experience greater social attention and possibly rejection for their deviation from health related social norms than boys do, translating into a greater perception of physical and social difficulty and lower scores on QOL measures.

This study has limitations. Disease complexity may contribute to nonsignificant results [35]. Operationalizing disease severity in terms of monostotic, polyostotic and McCune Albright syndrome, and pain in terms of frequency may have masked statistical associations. Further research using the FDFPR might seek to refine measurement of these constructs and make use of the number of bones participants report affected and the presence of specific endocrinopathies. Likewise, it might use pain intensity items from the BPI. Craniofacial involvement might also be differentiated in terms of visibility and/or functions specific to the region (sight, hearing, chewing and breathing) that may be affected by FD. The small sample size further lacks adequate power to reliably determine the importance of demographic and medical factors on QOL scores.

These results should not be interpreted as representative of all pediatric FD/MAS patients. The proportion of MAS patients in the sample is well above the estimated 5% of FD cases in the population, and the proportion of polyostotic cases in the sample is greater than monostotic cases. If disease seriousness is associated with QOL, this would tend to bias the sample toward worse scores on the PEDS-QL and HADS. While accessible to an international participation, the FDFPR is primarily comprised of individuals from the United States. The sample is not racially representative of the US population, a problem of other FD/MAS studies. It is also skewed to children of educated parents. Thus, mean scores on the PEDS-QOL and HADS could indicate lower levels of distress then exist in the population because the sample contains comparatively few individuals who lack access to reliable health care and/or face great social and economic obstacles for coping with a chronic illness.

The skewed demographics of the sample may reflect the greater resources that white, educated parents possess to locate the FDF and the FDFPR and to complete the lengthy battery of questions. It may also reflect a greater mistrust in medical research among members of minority populations that have been systematically neglected or damaged by prior medical research [45]. For the FDFPR to reach its potential to improve patient care, it must diversify the sample population. One avenue is to more actively recruit participants through pediatricians and general practitioners who treat underserved populations.

Research drawing from the FDFPR is further limited by its structure and scope. For example, this study uses the parent proxy of the PEDS-QL (ages 2–7) and the self-report of the PEDS-QL for ages 8–17 that are available. Because the format varies they may not be aggregated and it is not possible to ascertain whether age differences result in different scoring patterns on the PEDS-QL. Incorporating the self-report for lower ages and the proxy report for all ages would assist with resolving this issue. On the other hand, the online administration of measures in the FDFPR may encourage more accurate expressions of distress, because it reduces if not eliminates socially desirable responding [46]. The HADS was developed for in person administration and studies that have compared in person and remote administration of the HADS, via mail and online, have found higher scores in the remote samples [42, 46].

The PEDS-QL is a comprehensive measure that is valuable for detecting the clinical efficacy of treatment and is relatively short. To the extent that published norms exist for healthy children and chronically ill children, it might serve as a screening device to identify areas of trouble for individuals. Toward that end, it would be desirable to examine core scores, especially with younger children. The Total score encompasses the School Function core score, which the developers recommend only be used for descriptive or exploratory analysis in children seven and under.

The HADS was developed as a screening device and is limited to a narrow scope of depressive symptomology related to loss of the pleasure response. It does not assess feeling blue or sad and intentionally excludes some somatic symptoms [47, 48]. From the standpoint of understanding the impact of FD/MAS on quality of life, it could result in underestimating psychological distress, especially depressive symptoms, experienced by FD/MAS patients. The HADS has value in the treatment of FD/MAS as a diagnostic tool that leads to referral for further assessment and care and reasonably can be used in clinical appointments due to its brevity. Bearing in mind its limitations as comprehensive measure, it has value in the FDFPR because it enables comparison of the pediatric and adult populations of FD/MAS patients. Other measures should be used in research to assess the full extent of anxiety and depression in the pediatric FD/MAS population.

Participation in the FDFPR is not limited by treatment costs or the specific interests of treating clinicians. Thus, the FDFPR could become a database that encompasses patients receiving the full scope of treatments currently in use and that documents the full range of ways patients cope with their symptoms. The FDFPR could also be a venue for FD/MAS researchers to efficiently and cost effectively study new PRO measures. Toward this end, the FDFPR needs to focus on increasing participation of children and developing recruitment strategies to diversify the participant base.

## Conclusions

This study extends prior investigations of quality of life in children with FD/MAS using two instruments recommended by the International FD/MAS consortium. These results suggest that both age and gender may influence reported distress in ways not previously documented. Additionally, the results suggest the quality of life of pediatric FD/MAS patients may be more compromised than children with other chronic illnesses, suggesting a need to explore how unique qualities of the FD/MAS patient experience may result in disfunction and distress.

Monitoring the quality of life of pediatric FD/MAS patients is a desirable component of medical care. Early detection of problems in social interaction, management of emotions, in the school setting, and anxiety and depression as well as physical function will enable referral to appropriate resources. Simply raising this broad scope of issues with adolescents may increase their willingness to discuss social and psychological matters that they typically hold close and seek to manage on their own.

## Data Availability

The data that support the findings of this study are available from the Fibrous Dysplasia Foundation but restrictions apply to the availability of these data, which were used under license for the current study, and so are not publicly available. Data are however available from the authors upon reasonable request and with permission of the Fibrous Dysplasia Foundation.

## Abbreviations

BPI: Brief Pain Inventory
COPD: Chronic obstructive pulmonary disease
CFD: Craniofacial Fibrous Dysplasia
FD: Fibrous Dysplasia
FDF: Fibrous Dysplasia Foundation (now FD/MAS Alliance)
FDFPR: Fibrous Dysplasia Foundation Patient Registry (now FD/MAS Alliance Patient Registry)
FD/MAS: Fibrous Dysplasia / McCune Albright Syndrome
HADS: Hospital Anxiety and Depression scales
MAS: McCune-Albright Syndrome
NIH: National Institutes of Health
PEDS-QL: Pediatric Quality of Life Inventory
QOL: Quality of Life
2003HC: 2003 Healthy Child
